# Sex differences persist in visuospatial mental rotation under 3D VR conditions

**DOI:** 10.1371/journal.pone.0314270

**Published:** 2024-11-25

**Authors:** Oliver L. Jacobs, Katerina Andrinopoulos, Jennifer K. E. Steeves, Alan Kingstone

**Affiliations:** 1 Department of Psychology, University of British Columbia, Vancouver, Canada; 2 Department of Psychology and Centre for Vision Research, York University, Toronto, Canada; Pennsylvania State University, UNITED STATES OF AMERICA

## Abstract

The classic Vandenberg and Kuse Mental Rotations Test (MRT) shows a male advantage for visuospatial rotation. However, MRTs that have been adapted for use with real or physical objects have found that sex differences are reduced or abolished. Previous work has also suggested that virtual 3D objects will eliminate sex differences, although this has not been demonstrated in a purely visuospatial paradigm without motor input. In the present study we sought to examine potential sex differences in mental rotation using a fully-immersive 3D VR adaptation of the original MRT that is purely visuospatial in nature. With unlimited time 23 females and 23 males completed a VR MRT designed to approximate the original Vandenberg and Kuse stimuli. Despite the immersive VR experience and lack of time pressure, we found a large male performance advantage in response accuracy, exceeding what has typically been reported for 2D MRTs. No sex differences were observed in response time. Thus, a male advantage in pure mental rotation for 2D stimuli can extend to 3D objects in VR, even when there are no time constraints.

## Introduction

Mental rotation ability is a widely researched construct in psychology that is typically measured using either the Shepard and Metzler paradigm [1] or the Vandenberg and Kuse Mental Rotations Test (MRT) [2]. Shepard and Metzler’s computer-displayed test consists of a pair of floating cube-based blocks with one rotated to a new orientation and the pair either being congruent or incongruent in shape. Participants are scored on how quickly and how accurately they categorize the pair of shapes as matching or mismatching. Vandenberg and Kuse’s Mental Rotations Test (MRT) [2] requires participants to view a target line drawing composed of articulated blocks before indicating which of four other drawings match the target. Two drawings depict the target in different spatial orientations while the other two are foils. These tests are also what is sometimes referred to as “quasi-3D” as they depict 3D shapes but are presented in 2D either on a computer screen or on paper [3].

Since the emergence of these tests, large sex differences have been reported with males tending to score higher than females [4, 5]. A number of explanations for these differences have been put forward ranging from distinct biological origins [6, 7] to contrasting response strategies [8, 9]. When mental rotation adaptions of these tests have used real or virtual 3D objects, the sex difference has typically either been reduced or completely abolished (but see [10] for a null effect of a 3D format).

An early example of the impact of a 3D format was provided by McWilliams et al. [11] who found that sex differences disappeared when using an adaptation of the MRT wherein participants were asked to discriminate between real 3D objects rather than 2D images of 3D objects. Robert and Chevrier [12] later found the same pattern of results, again using real objects as did Heil and Jansen-Osmann [13]. More recently, Fisher et al. [14] found that the use of real objects reduces the sex effect suggesting that at least part of the sex difference on the MRT is a consequence of whether the objects are real or merely 2D depictions of real 3D objects.

Parsons et al. [15] in a seminal paper investigated sex differences using virtual objects in a VR adapted MRT. Parsons and colleagues used an Immersadesk system, which by today’s standard provides crude levels of virtual immersion because of its comparatively poor field of view and visual fidelity. Nevertheless, the stimuli were presented in 3D thereby affording additional perceptual depth cues that are not available with 2D images, namely stereopsis and motion parallax. Note that these virtual objects are not real objects that can be interacted with, nor do they convey the same affordances as real objects—in particular vergence and accommodation cues [16]. Still, sex differences were abolished, suggesting virtual objects were similar enough to real objects that they too negate the sex effect.

However, there are several other reasons why Parsons et al. [15] may have failed to find a sex difference. Most concerningly is that the VR task itself was not purely visual in nature. Participants were asked to manually rotate a virtual object to the same spatial orientation as the previously seen target object. Thus, the VR task involved a component of visuomotor rotation. Specifically, engaging visuomotor systems may have reduced sex differences by introducing strategies to solve the MRT that would not be possible in a purely visual version of the task. This possibility is supported by the fact that motor systems play a crucial role in facilitating cognition, particularly regarding mental rotation ability [17, 18].

Furthermore, it is worth highlighting that the findings in the Parsons et al. [15] investigation reflect a failure to reject the null hypothesis of no sex difference. Reasoning scientifically from a null hypothesis is a risky endeavor because, for example, p-values can be highly misleading about the amount of evidence supporting the null hypothesis. Indeed, research has shown that the difference between the actual amount of evidence for the posterior probability and the evidence suggested by the p-value can be as much as an order of magnitude [19].

Surprisingly, since the Parsons et al. [15] investigation there has been spare work investigating sex differences using a VR version of the MRT. One past study [20] merely used VR to depict 2D versions of the stimuli—essentially replicating the Vanderberg and Kuse [2] test, but substituting paper for a digital version of the stimuli. More notably, a recent pilot study [21] found a medium-sized sex difference in favour of males in a 3D VR MRT but the effect failed to pass a significance threshold providing limited evidence for either the null or alternative hypothesis.

In sum, when MRTs are conducted with 2D representations of 3D objects, a sex difference is obtained. With real 3D objects the MRT sex differences are reduced or eliminated. There is some preliminary evidence to suggest that sex differences are also eliminated when objects are rendered as 3D in VR [15, 21] but the evidence of this finding is not conclusive. What is clear is the need to better understand sex differences in mental rotation as there is a growing interest in training spatial skills (including mental rotation) at a young age given links between spatial skills and later performance in the domains of Science, Technology, Engineering, and Mathematics (STEM) [22–25]. This link is particularly apparent for certain fields such as chemistry where spatial thinking is required for understanding concepts like isomers and chirality [24]. Moreover, VR is increasingly being used as an educational tool [25] and if sex differences do extend to mental rotation in VR, then training interventions at a young age might be used to reduce gaps in spatial abilities.

The aim of the present study is to address this issue regarding VR. We measured male and female response accuracy, and response speed, in a purely visual version of the Vandenberg and Kuse [2] MRT with objects rendered as 3D in VR. Finally, it is worth noting that we administered the MRT without a time limit, as time pressure has been shown to accentuate MRT sex differences [5].

## Methods

### Participants

The opportunity to volunteer for this study was delivered through an online service that is made available to the Human Subject Pool at the University of British Columbia in the Department of Psychology. The title of the study was benign, making no mention of its intent or research methods. 46 students (23 female, mean age = 22.7 years; 23 male, mean age = 22.3) took part for course credit or $5.00 remuneration. A G*Power test with the observed effect size yielded a power estimate of 99.1%. Similarly, a post-hoc bootstrapping analysis conducted using the R package *WebPower* [27] estimated power to be 99.7% given the observed means, standard deviations, skews, and kurtoses with 10000 iterations. All participants were required to have normal or corrected-to-normal vision. Participants provided written consent before the start of the experiment. The study was approved by the ethics board of the University of British Columbia (H10-00527).

### Equipment

The experiment was conducted using an HTC Vive VR head-mounted display controlled by a custom-built desktop computer (PC with Intel i7-8700K CPU @ 3.70GHz, 32 GB RAM, Nvidia GeForce GTX 1080 Ti, 1TB Samsung SSD). The HTC Vive headset (Vive model 2PU6100) has a resolution of 1080 x 1200 pixels per eye with a 110-degree horizontal field of view, a 113-degree vertical field of view, a refresh rate of 90 Hz, and a weight of 635 grams. The experiment was built using Unity (version 2019.1.7f1), which is a popular engine for designing 3D environments and games. Participants used the HTC Vive controllers which offer point-and-click functionality in order to make responses.

### Stimuli

**Fig 1:**
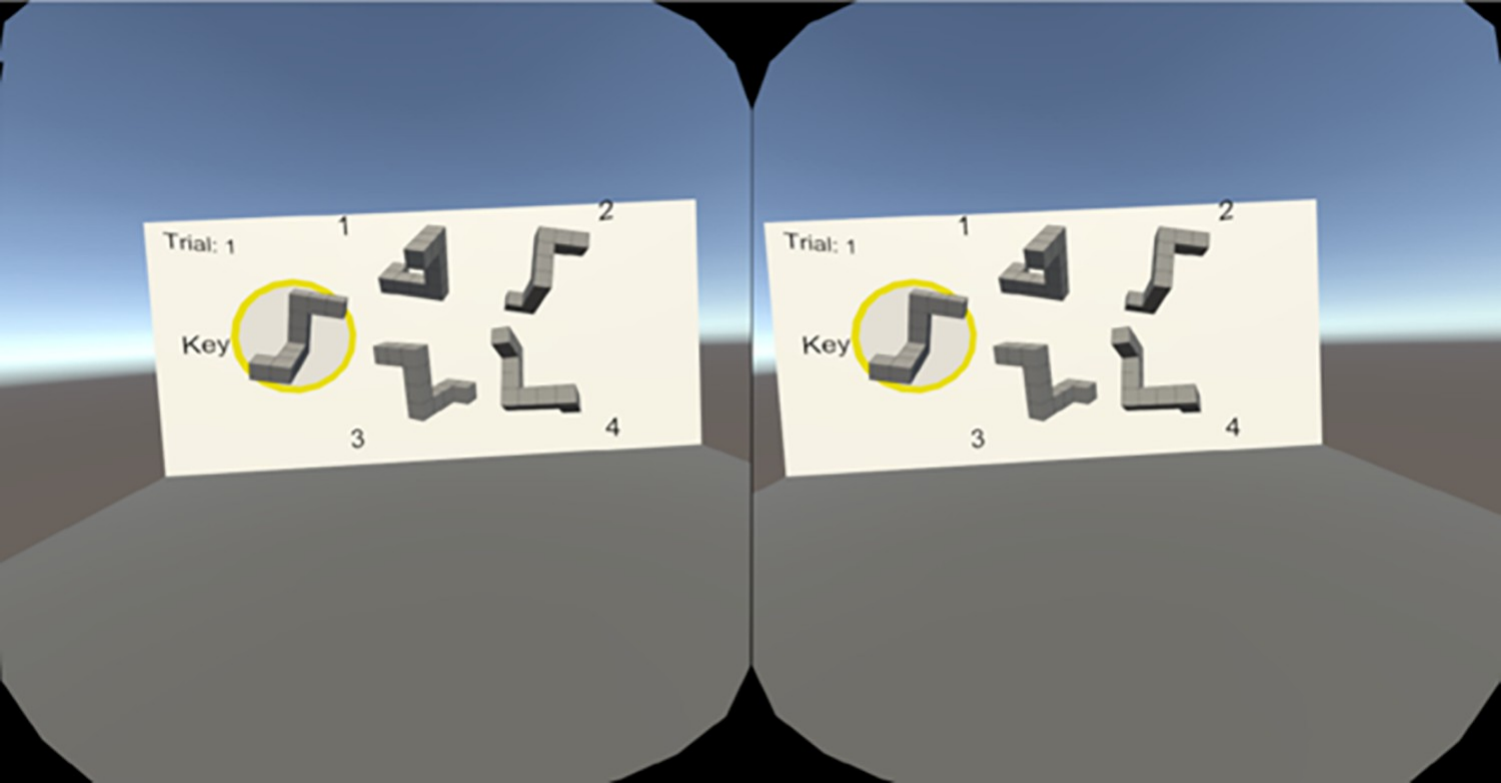
A screenshot of stereo vision afforded in the VR head-mounted display. The image on the left was displayed to the left eye and the image on the right was displayed to the right eye. Note that the stimuli appear as 2D blocks because the image is a 2D reproduction of the VR scene.

**Fig 2:**
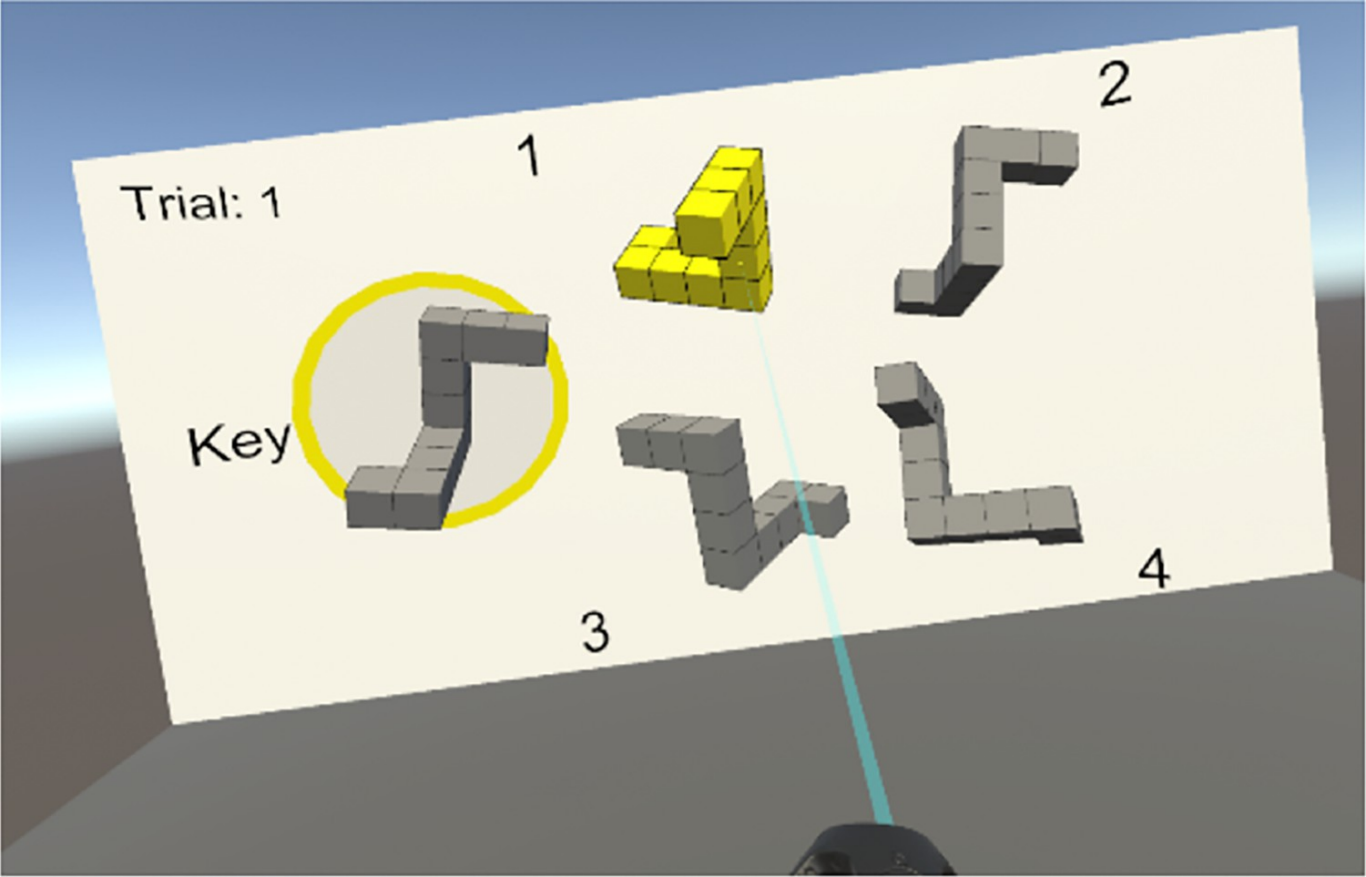
The VR adaptation of the stimuli for trial 1. The image is a monocular picture from the VR task. The target, or key stimulus, is circled in yellow. Participants are asked which two of the four exemplars to its right are the same as the target. The participant here has indicated object 1 as matching the key which renders it yellow.

The 3D block stimuli used a best-approximation protocol based on the stimuli developed by Vandenberg and Kuse [2]. The stimuli were virtual objects floating in an environment consisting of a grey floor, a white wall behind the blocks, and a blue sky. On the wall, the current trial number and a yellow circle indicating the current key block were displayed. The blocks were approximately 1 x 1 meter in width/height and were viewed from 10 meters away (subtending approximately 5.7 degrees of visual angle). While stereopsis would likely not have been a helpful depth perception cue at these distances, there was some degree of stereopsis (see Fig 1). Motion parallax feedback was present and participants were able to move their head freely. Fig 2 shows the original MRT stimuli and a 2D representation of a corresponding trial with virtual objects. Note that the VR environment provides enhanced ecological validity of the stimuli without sacrificing experimental control (e.g., consistent lighting and shading, as well as precise chronometric measurement).

### Procedure

Upon arrival at the lab, participants were given an informed consent form and a document containing broad information about the study (e.g., our interest is to understand how people perceive items in a virtual world). It made no mention of sex differences. Participants were familiarized with the VR equipment and given oral instructions on how to use the controller to select answers that matched the key. Participants also viewed the same instructions in virtual reality upon the initiation of the program. Participants were instructed to remain seated during the experiment but they were free to move their head and body if they chose to do so as long as they remained seated. There was a total of 20 trials, presented randomly in order to control for order effects, with each trial having 2 correct answers. The participants were given unlimited time to complete all of the trials, without feedback and would move to the next trial after selecting their second answer. After finishing the 20 trials, participants were given a survey while still in VR. The survey asked participants to indicate their sex and age. After completing the survey, participants removed the headset before being debriefed.

### Data analysis

Proportion correct was calculated by taking the total number of trials with 2 correct answers and dividing by the total number of trials (20). Analyses were also conducted by measuring proportion correct as the number of correct answers out of the total number of correct answers (40). This did not change the significance of the following results. Total response time was recorded as the length of time between the end of the first trial and the completion of the task. The first trial was not included in the total response time as it included the time getting familiar with the VR environment and controls. We used a number of R packages: *papaja* for manuscript creation [26], Webpower for power analysis [27], R.Core for the Z-test, *survival* for the Cox proportional hazards regression [28], and *beeswarm* for figures [29]. We also used two packages for data cleaning: *dplyr* by [30] and *data*.*table* [31]. The data are available on OSF at: DOI 10.17605/OSF.IO/WAP4C.

## Results

**Fig 3:**
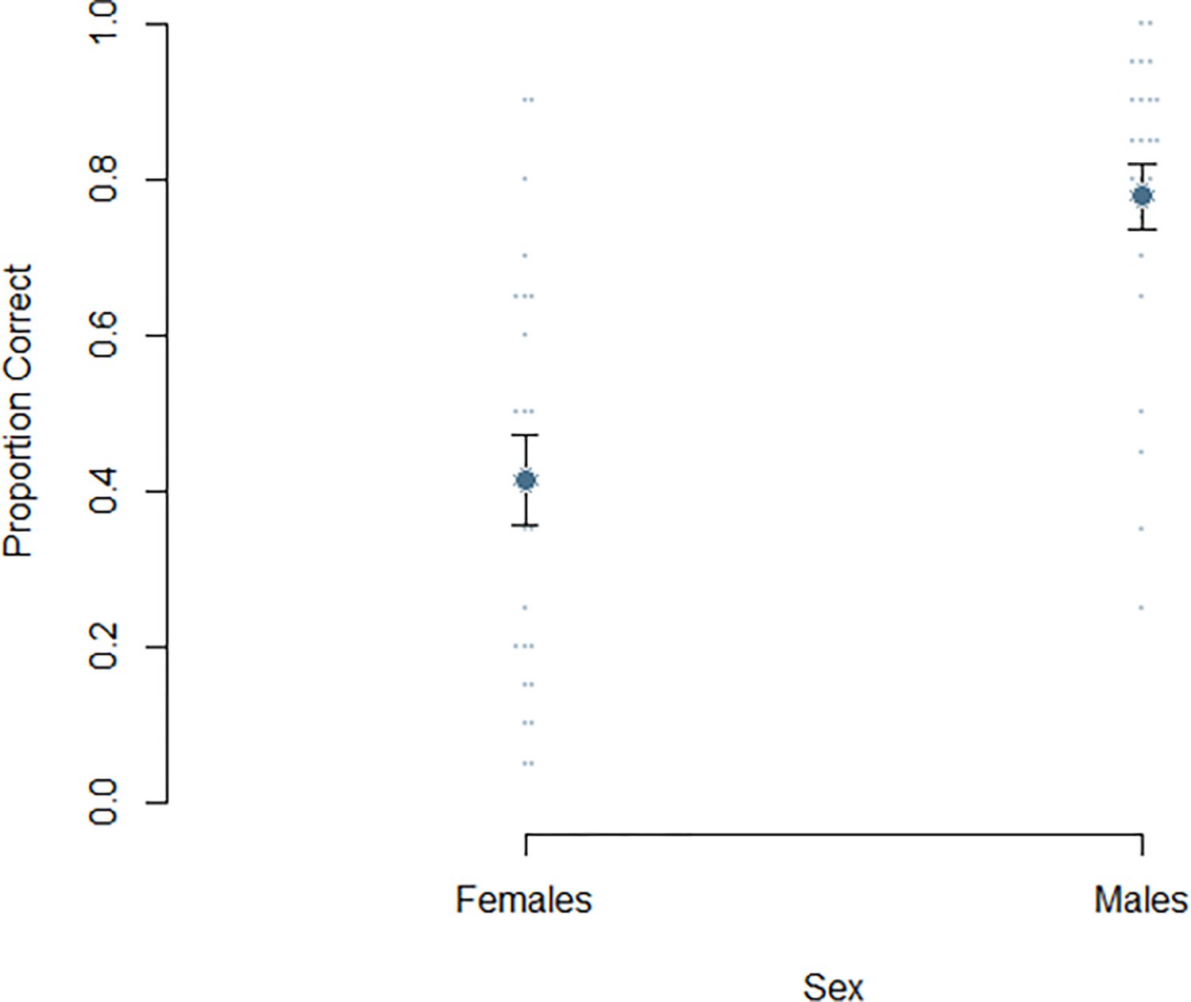
A plot depicting proportion correct as a function of sex. Dots indicate individual values. Error bars represent standard error.

**Fig 4:**
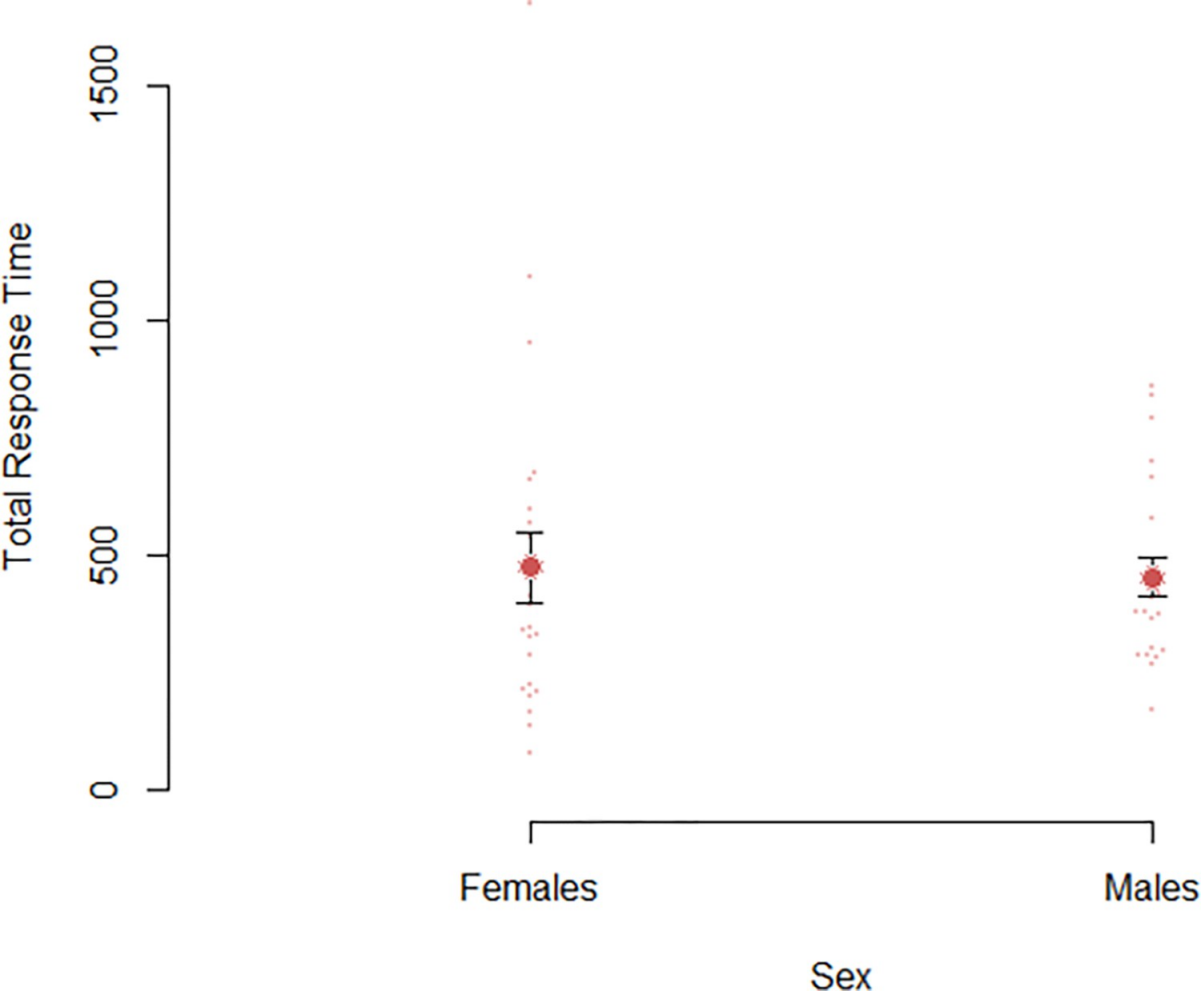
A plot of total response time as a function of sex. Dots indicate individual values. Error bars represent standard error.

The primary question of interest concerned whether there were significant differences in mental rotation accuracy between males and females. A Z-test of proportions revealed that males were significantly more accurate compared to females, Z = 11.29, *p* < .001 (Fig 3). This corresponds to a large effect size, *h* = 0.764. We also wanted to investigate whether there were differences in the length of total response times (Fig 4). A Cox proportional hazards model was employed to examine the effect of sex on the total time taken to complete all questions. The hazard ratio for males compared to females was 1.079, 95% CI [0.590, 1.975], *p* = .804. The concordance statistic was 0.47 (SE = 0.044), which indicates a poor fit of the model to the data. All associated tests, including the likelihood ratio test, Wald test, and the score (log-rank) test supported the non-significance of sex as a predictor for time to completion (*p*’s > = .800).

## Discussion

Past studies using the Mental Rotations Test, which contain 2D representations of 3D objects, have routinely found large sex differences in mental rotation [4]. When participants have been asked to mentally rotate more realistic stimuli such as real 3D objects, the sex effect has typically either been reduced or eliminated entirely [11–14]. The reports of a comparable finding for virtual objects [15, 21] are compromised by confounding factors, such as the failure to isolate visual mental rotation from the contribution of visuomotor processes and reasoning from null findings. The present study addressed the limitations of the previous work [15, 21] to test if sex differences are eliminated when individuals are asked to perform a purely visual MRT with 3D virtual objects in a modern high-fidelity head-mounted display.

The results were clear. Males scored higher than females on the MRT with virtual 3D objects, replicating what is routinely found when individuals perform a 2D MRT [4]. Note that we obtained this sex difference despite the fact that our MRT was given without a time limit, which controls for task time requirements that may artificially create or inflate sex differences [5]. Collectively, the fact that we found a sex effect with virtual objects, effectively replicating the effect found routinely with a 2D MRT, suggests that the findings from a 2D MRT can generalize to virtually displayed 3D objects; and that the MRT with virtual 3D objects does not generalize to a MRT with real objects. In sum, while virtual objects are more realistic and afford more perceptual cues than 2D images, virtual objects, unlike real objects, do not reliably abolish or reduce the magnitude of sex differences in an MRT. Our results support the concerns we raised with the two previous studies using a VR-adapted MRT, one of which failed to find an effect in a design that was not purely visual [15] and one of which failed to use 3D virtual objects [21].

### Future directions

Following our findings, several outstanding issues remain to be examined. A male experimenter, for example, was used to collect data from all the participants in the present study, which may have invoked a stereotype threat [32] for some of the female participants—though we hasten to add that a recent meta-analysis suggests that stereotype threat is not a significantly deleterious effect in spatial cognition tasks between sexes [33]. The use of VR may be experienced differently by each sex especially as VR becomes more adopted by male gamers in particular [34]. While there is no evidence that the novelty of VR alone could create a sex difference in mental rotation, it is possible that it may have accentuated any differences. Future research could ascertain previous experience with video games and VR, in addition to the role of headset weight relative to body weight, to determine to what extent, if any, these variables might have contributed to the large sex effect size. There are also concerns about the degree to which the Vandenberg and Kuse MRT is a pure measure of mental rotation as some strategies can involve using feature-based differences between shapes to solve some trials without having to mentally rotate the blocks [9]. Other variations of mental rotation tasks in 3D VR environments would test the generalizability of the present findings. Other limitations are more specific to present study: the VR environment participants took part in was deliberately sparse to avoid extraneous visual distraction, but as a result, it was unlike many everyday environments. Future studies could use a visually richer environment such as a 3D model of a classroom, in addition to including a greater number and more diverse range of participants in terms of age and demographics.

### Conclusion

VR has immense potential for moving forward important questions related to how the realism of stimuli influence sex differences in mental rotation. This is especially timely given recent interest in how sex differences may be an artifact of the artificiality of the stimuli (see [14]) and other ideas related to *how* the MRT is presented affects sex differences. Modern 3D VR tools enable precise manipulation of stimuli features and perceptual cues like lighting, shading, stereopsis, and motion parallax. In addition, VR allows researchers to collect additional signals that can help inform the scientific understanding of mental rotation. For example, both head and eye movements have been shown to play a critical role in the facilitation of cognitive abilities, especially in mental rotation, yet they have not been investigated together in a VR paradigm. In addition, there is a growing interest in using VR as an educational tool to provide training in spatial thinking and ability as early spatial skills have been linked to later STEM performance [24, 25]. The present findings suggest that the sex differences found in traditional MRT tasks extend to VR and therefore may be targeted for intervention to reduce sex differences and ideally improve later STEM performance for all users. VR can provide a controlled, systematic, yet highly engaging medium for these interventions.

Finally, sex differences continue to be an important area of research because understanding how the perceptual and cognitive functions differ between males and females can help to shed light on neurological, developmental, psychiatric, and psychological disorders that differentially affect the sexes. Our findings suggest that VR is highly sensitive to sex differences in mental rotation when assessed in a purely visuospatial task. In sum, we have discovered that the findings routinely found for a 2D MRT can extend to, and replicate within, a 3D VR environment.
